# Peripheral Nerve Stimulation: The Evolution in Pain Medicine

**DOI:** 10.3390/biomedicines10010018

**Published:** 2021-12-23

**Authors:** Alaa Abd-Elsayed, Ryan S. D’Souza

**Affiliations:** 1 Department of Anesthesiology, University of Wisconsin, Madison, WI 53711, USA; 2 Department of Anesthesiology and Perioperative Medicine, Division of Pain Medicine, Mayo Clinic Hospital, Rochester, MN 55905, USA; dsouza.ryan@mayo.edu

## 1. History of Peripheral Nerve Stimulation

Electrical stimulation of peripheral nerves has been utilized for a variety of indications for over five decades. It was first described by Shelden and colleagues in 1966, [1] who reported that implanted peripheral nerve stimulation (PNS) electrodes delivering stimulation at 14,000 hertz (Hz) was associated with temporary pain suppression in trigeminal neuralgia. Subsequently in 1967, a case series [2] of eight patients with chronic cutaneous and neuropathic pain achieved pain suppression when the PNS device was turned on. Historically, the approach for PNS implantation involved the surgical dissection of tissues to visualize the nerve and placement of the PNS lead adjacent to the nerve [3]. This was modified to involve the placement of a transposed fascial graft between the PNS lead and the targeted nerve, although this approach fell out of favor due to surgical complexity and complications [4]. The concept of percutaneous PNS lead placement was introduced in 1999 by Weiner and colleagues, who reported that PNS lead placement performed via a percutaneous approach can offer analgesia in patients with occipital neuralgia [5].

Unfortunately, earlier studies highlighted only modest efficacy along with concerning complications, such as lead erosion, lead migration, PNS-related fibrosis, and nerve injury [6,7]. With the advent of spinal cord stimulation (SCS) and the associated high-quality evidence revealing impressive outcomes in medically intractable pain syndrome, PNS was viewed by many physicians as a less desirable option. Despite these data from earlier published studies, considerable technological advancements within the past decade in PNS devices have revolutionized the practice of pain medicine. The application of electrical stimulation of peripheral nerves has also impacted other fields besides pain medicine, including testing for neuromuscular conduction in anesthesia and intensive care, phrenic nerve stimulation in diaphragmatic palsy, motor nerve stimulation in patients with hemiplegia or paraplegia, vagal nerve stimulation in patients with epilepsy, and autonomic stimulation in disorders of the gastrointestinal and genitourinary systems.

## 2. Current Indications for Peripheral Nerve Stimulation

Technological innovations have been achieved to facilitate PNS placement via a percutaneous approach, performed in ambulatory practices instead of an open neurosurgical placement to improve implantation and imaging techniques with ultrasound guidance, incorporate hardware miniaturization and external implantable pulse generators (IPG), and reveal high-quality data on safer placement and reduced adverse events. Unlike SCS, PNS offers greater selectivity and accuracy by targeting well-defined neuronal targets. Novel design-specific and minimally invasive PNS modalities comprise either fully implantable leads (e.g., StimRouter, Bioness Inc., Valencia, CA, USA; StimQ, Stimwave Inc., Pompano Beach, FL, USA) and temporary externalized leads (e.g., SPRINT, SPR Therapeutics Inc., Cleveland, OH, USA) [8]. Furthermore, these percutaneous leads may either utilize no implantable IPG or may utilize a hybrid system consisting of an external power source with an internal generator.

To date, there has been Food and Drug Administration (FDA) approval for temporary PNS up to 60 days for the treatment of chronic pain, post-surgical pain, and post-traumatic pain of the back and/or extremities [9]. This approval was based on findings from several feasibility studies [10,11,12,13] and a randomized controlled trial by Gilmore and colleagues [14,15]. Recently, in October 2021, the FDA has also expanded the use of temporary PNS for facial/head pain, allowing on-label use for the stimulation of occipital nerves. Importantly, 60-day PNS with temporary externalized leads is associated with long-term analgesic benefits that are sustained at 12 months [13,14]. Other manufacturers with permanent PNS implants also offer PNS technology that is FDA-approved and Conformité Européenne (CE) approved for the treatment of peripheral mononeuropathy that is not responsive to the best medical management.

Interestingly, PNS has also been utilized for complex regional pain syndrome (CRPS) and phantom limb pain. This suggests that pain manifestations that are centrally mediated may be treated with PNS, and not just centrally mediated therapies such as dorsal column SCS or dorsal root ganglion SCS.

## 3. Mechanism of Peripheral Nerve Stimulation

While the mechanism of PNS remains unknown, many suggest that both peripheral and central mechanisms exist [16]. Central mechanisms include inhibition of pain signals from inhibitory dorsal horn interneurons (Gate Control Theory), modulation of wide dynamic range (WDR) neurons in the dorsal horn, and reduction of neural impulses via the medial lemniscal pathway. By inhibiting WDR neurons in the dorsal horn, as well as reducing activity of A-β fibers in the medial lemniscal pathway, PNS may decrease the degree of central sensitization and hyperalgesia [16,17]. Furthermore, in the spinal cord, PNS may affect biochemical pathways, including serotonergic, GABAergic, and glycinergic pathways [18]. Peripheral mechanisms include disruption of nociceptive afferent fibers, alteration of local microenvironment, and downregulation of inflammatory mediators. The number of ectopic discharges may also decrease with the application of PNS [19].

## 4. Future Directions

Currently, PNS is more heavily utilized in localized pain conditions limited to one or two peripheral nerve distributions. As technology evolves, we propose that more diffuse pain manifestations such as pain involving the entire upper or lower extremity, or localized pain in a stocking glove distribution, may potentially be amenable to treatment with PNS. This may also pave the way for additional FDA-approved and CE-approved indications for PNS devices.

Another important technological piece that warrants further investigation is analgesic outcomes based on a type of PNS waveform delivery and electrical programming variables (frequency, pulse width, intensity). The field of SCS therapy has witnessed the development of multiple waveforms including tonic, 10-kHz, burst, and differential target multiplexed (DTM). Typically, PNS technology delivers low-frequency and tonic stimulation that stimulates sensory fibers. Other waveforms such as paresthesia-free, 10-kHz PNS have been reported in observational studies, yet the bioelectronics principles that govern waveform modalities are still in their infancy in clinical practice [12]. Novel waveforms not only allow for unique variation of stimulation that is better suited for the patient’s particular pain condition, but also allows the implanting provider to adjust settings for patient comfort and offers an option for salvaging pain control by transitioning from a less effective waveform to a new waveform that the patient has not previously trialed. Efficacy of PNS may also be impacted by distance of leads from the target nerve, tissue impedance, consistency and duration of PNS delivery, lead shape, and electrical field. Ultimately, these bioelectronics effects from PNS continue to be elucidated in studies.

Temporary PNS for 60 days may offer improved long-term analgesic outcomes over a 12-month period. Studies should elucidate the central mechanisms that may mediate these long-term effects. The authors also query if combination stimulation with PNS, SCS, and/or cortical stimulation simultaneously may offer additive or synergistic therapeutic effects. Other applications for PNS, such as targeting peripheral nerves implicated in general inflammation, myocardial ischemia, arrhythmias, post-traumatic stress disorder, and anxiety, are also high-priority areas warranting investigation.

In conclusion, PNS is well-positioned to revolutionize the field of pain medicine as approved indications are expanded, unique waveform modalities are developed, and stimulation parameters are optimized and personalized to the patient’s needs. PNS may be an effective modality and alternative pharmaceutical intervention.

## Data Availability

Not applicable.
